# Reduction in HIV community viral loads following the implementation of a “Treatment as Prevention” strategy over 2 years at a population-level among men who have sex with men in Hangzhou, China

**DOI:** 10.1186/s12879-017-2927-2

**Published:** 2018-02-01

**Authors:** Lin He, Jiezhe Yang, Qiaoqin Ma, Jiafeng Zhang, Yun Xu, Yan Xia, Wanjun Chen, Hui Wang, Jinlei Zheng, Jun Jiang, Yan Luo, Ke Xu, Xingliang Zhang, Shichang Xia, Xiaohong Pan

**Affiliations:** 1 Zhejiang Provincial Center for Disease Control and Prevention, No. 3399 Bin Sheng Road, Binjiang District, Hangzhou, Zhejiang Province People’s Republic of China; 2 Hangzhou Center for Disease Control and Prevention, Hangzhou, Zhejiang China

**Keywords:** Men who have sex with men, Treatment as prevention, Antiretroviral therapy, Community viral loads

## Abstract

**Background:**

Previous studies have shown that the increased coverage of antiretroviral therapy (ART) could reduce the community viral load (CVL) and reduce the occurrence of new HIV infections. However, the impact on the reduction of HIV transmission among men who have sex with men (MSM) is much less certain. The frequency of HIV infections in MSM have been rapidly increasing in recent years in Hangzhou, China. The “Treatment as Prevention” strategy was implemented at a population-level for HIV-infected MSM from January 2014 to June 2016 in Hangzhou; it aimed to increase the ART coverage, reduce the CVL, and reduce HIV transmission.

**Methods:**

We investigated a subset of MSM diagnosed with HIV pre- and post-implementation of the strategy, using random sampling methods. Viral load (VL) testing was performed for all enrolled individuals; the lower limits of detection were 20 and 50 copies/mL. The data on infections were collected from the national epidemiology database of Hangzhou. Logistic regression analyses were conducted to identify factors associated with the differences in social demographic characteristics and available VL data.

**Results:**

The ART coverage increased from 60.7% (839/1383) during the pre-implementation period to 92.3% (2183/2365) during the post-implementation period in Hangzhou. A total of 940 HIV-infected MSM were selected for inclusion in this study: 490 (52.1%) and 450 (47.9%) MSM in the pre- and post-implementation periods, respectively. In total, 89.5% (841/940) of patients had data available on VL rates. The mean CVL was 579 copies/mL pre-implementation and this decreased to 33 copies/mL post-implementation (Kruskal-Wallis < 0.001). The mean CVL decreased for all variables investigated post-implementation of the treatment strategy (*P* < 0.05). The undetectable VL (≤400 copies/mL) rate pre-implementation period was 50.0% which increased to 84.7% post-implementation (*P* < 0.001). The mean CVL at the county level significantly decreased in each county post-implementation (Kruskal-Wallis < 0.05).

**Conclusion:**

Our study confirmed a population-level association between increased ART coverage and decreased mean CVL; overall 84.7% of HIV infected MSM had an undetectable VL and were no longer infectious.

## Background

The Joint United Nations Programme on HIV/AIDS implemented the “90–90-90 strategy” in 2014 with the aim of ending the HIV epidemic; the strategy aims to achieve 90% diagnosis, 90% treated, and 90% virally suppressed for those living with HIV by 2020 [1]. The HIV Prevention Trials Network (HPTN) 052 trial [2] found that using antiretroviral therapy (ART) could lead to a reduction in viral loads (VL) for HIV-infected individuals, thus leading to a 96% reduction in heterosexual HIV transmission in stable HIV-discordant couples. However, the magnitude of individual-level treatment on the reduction in HIV transmission among men who have sex with men (MSM) is much less certain [3]. MSM usually have multiple sexual partners and unprotected anal intercourse [4]. A previous meta-analysis showed that relatively small changes in sexual risk behavior could overpower any benefits of ART [5], given the higher transmission probabilities associated with unprotected anal sexual acts; thus, ART might be less effective in preventing transmission among MSM [6]. Even though data have not proven that ART will reduce HIV incidence and new infections at the population-level among MSM, the impact of treatment as a preventive strategy to reduce the frequency of new infections is controversial.

ART has been proven to reduce the VLs of infected individuals, and might reduce the risk of sexual transmission to uninfected persons [2]. Community viral load (CVL) is defined as the mean or total HIV VL of infected individuals in a given geographic area or population [3, 7]. Mean and total CVLs are indicators of the population’s viral burden and have been utilized as useful measures for assessing ART and tracking its impact on transmission [8–10]; they have also been useful in assessing trends in local HIV/AIDS epidemics [11]. CVLs could be used as robust markers of the efficacy of “Treatment as Prevention” strategies [12]. Several studies have confirmed that elevated CVLs are associated with the incidence of new HIV infections in a population [13, 14]. The increased incidence of HIV is significantly associated with VLs; high VLs are strongly associated with new cases of HIV and the reduction in CVLs leads to a reduction in new HIV infections [7, 14, 15]. A study conducted in San Francisco, showed that decreases in the CVLs were accompanied by reductions in the frequency of new HIV infections [7]. Consistent with the CVL trends in San Francisco [16], the CVLs in South Carolina gradually decreased from 2004 to 2013 as did the number of new HIV infections. Similar to findings from San Francisco, data from India [10] showed undetectable VLs were the strongest correlates of HIV incidence in MSM [17]. Administrative data from Canada [18] and France [12] showed significant associations between increased highly active antiretroviral therapy (HAART) coverage and decreased CVLs with the frequency of new HIV infections. In contrast to findings from the studies conducted in San Francisco, South Carolina, and South Africa, a study in Columbia found no association between the trends in the mean CVL (mCVL) and the frequency of newly diagnosed HIV cases [11]. However, an HIV epidemic model demonstrated that mCVL was not necessarily a strong predictor of HIV incidence [19]. Another study from southern Alberta showed that increased HAART coverage did not reduce the CVLs or the frequency of new HIV diagnoses [20]. Additionally, in Australia [21], an observational database showed the decreased VLs were associated with increased HIV incidence.

In China, the frequency of MSM is exponentially growing; the sentinel surveillance of MSM conducted from 2010 to 2016 showed the HIV prevalence increased from 5.7 to 7.8% [22]. In Hangzhou, which is located in one of the more developed regions of China, the annual numbers of newly diagnosed HIV individuals are rapidly increasing; MSM account for approximately 60% of the new HIV infections. A respondent-driven sampling survey conducted in Hangzhou in 2014 showed the HIV prevalence among MSM was approximately 8.5% [23]. The “Treatment as Prevention” strategy was not implemented until 2014; thus, the impact of this strategy in China is unclear, especially in MSM. In order to control the HIV epidemic and reduce the incidence of new HIV infections, we implemented the “Treatment as Prevention” strategy from January 2014 to June 2016 in Hangzhou. Using this strategy, all MSM infected with HIV were encouraged to initiate ART, regardless of their CD4 count, in order to reduce the CVL and control the incidence of new HIV infections at the population-level among MSM. The aim of this study was to assess ART coverage, mCVL, HIV incidence, and frequency of new HIV infections from January 2014 to June 2016 pre- and post- the implementation of the “Treatment as Prevention” strategy among HIV-infected MSM in Hangzhou, China.

## Methods

### Study subjects

Individuals were considered for inclusion in this study if they were older than 18 years of age, residents in Hangzhou for over 3 months, HIV-infected MSM, and if they belonged to the follow-up management group of Hangzhou epidemiologic database, regardless of whether they were in care or lost to follow-up.

### Data collection

The data on infectious diseases were collected from the national epidemiologic database of Hangzhou, which tracks everyone who is diagnosed with HIV in China. The following data were collected: date of HIV diagnosis, education and other social demographic characteristics, CD4 counts, transmission route, and routine follow-up and treatment information. The above databases have been described in detail in previous publications [24, 25]. Prospective data were collected on patients who required VL testing in this study. Additional data on HIV incidence and the frequency of new infections were collected from surveillance databases.

### Study sampling and design

From the Hangzhou epidemiological data base, a random sampling method was used in order to select HIV-infected MSM to be included in the study; patients’ ID numbers in the epidemic database were sorted, and research subjects were randomly selected based on the sample size calculations. The pre-implementation period data were collected from the national epidemiologic database (data until the end of December 2013), while the post-implementation data were collected from the database (data until June 2016). This was a prospective intervention study. From January 2014 to June 2016, the “Treatment as Prevention” strategy was implemented in Hangzhou; the goal of this strategy was to increase ART coverage to 90% among HIV-infected MSM and decrease the population-level CVLs by 50% by June 2016. Random sampling methods were used to select MSM individuals with HIV infection in Hangzhou from before January 2014 (pre-implementation period) and after June 2016 (post-implementation period). The sample size was calculated using WINPEPI (PEPI- for-windows) version 9.5 software. The sample size calculations were performed based on the increased ART coverage rate and decreased mCVL, which would achieve 90% power to detect significant mean differences in empowerment scores at a level of 0.05. The minimum sample size required was 400. Data were prospectively collected from other surveillance databases in order to ascertain trends in HIV incidence and frequency of new infections.

### VL testing

VLs were not tested for enrolled patients on ART who had a VL test conducted within the past 6 months. For such patients the previous VL results were used for this study. Patients not on ART or those on ART whom did not have VL tests performed in the past 6 months, had VL testing completed by study investigators. Two sensitive polymerase chain reaction (PCR) techniques for measuring VLs were used in this study. The lower limits of detection were 20 and 50 copies/mL for these techniques, which were the same as those for patients with VLs completed in the past 6 months. Blood sampling was completed within a month after the completion of the selection of the HIV-infected MSM subjects for enrollment in the pre- (before January 2014) and post-implementation (after July 2016) periods.

### Mean community viral load calculation method

The mCVL was defined as the mean VL of HIV-infected MSM in Hangzhou. The Log10-transformed value was calculated according to the individual VL value, and then the arithmetic mean was calculated. Arithmetic mean log10-transformed individual VLs are approximately normally distribution. When calculating the mean value, we used half of the detection limit to calculate the VL below the detectable limit. Undetectable VLs were defined as an individual mean of ≤400 copies/mL due to the variation in the lower limits of detection using different testing platforms.

### HIV incidence and new HIV infections

The investigations of HIV incidence and new infections were conducted in the first halves of 2014 and 2016 for the pre- and post-implementation periods, respectively. The HIV incidence was calculated using the sentinel surveillance data for MSM in Hangzhou; all of the positive infections were detected using the BED capture enzyme immunoassay (BED-CEIA), and were used to calculate HIV incidence. New HIV infections included newly diagnosed HIV cases in 2014 and 2016 using BED detection with CD4 tests conducted within 3 months of diagnosis, showing CD4 counts ≥600 cells/μL [26]. BED-CEIA successfully detected 100% of newly diagnosed cases, and the CD4 within 3 months of diagnosis detected 97.0 and 96.4% of new cases in the pre- and post-implementation periods. All the newly diagnosed HIV cases were required to provide their current address in Hangzhou.

### Statistical analysis

Categorical variables are presented as frequencies and proportions while continuous variables are presented as medians and interquartile ranges (IQR). Differences in the general demographic characteristics were calculated using the Student’s T test or chi-squared (χ2) and Kruskal-Wallis tests. We determined the mean (standard deviation [SD]) and median (IQR) for mCVL. Logistics regression analyses were used to identify the factors associated with the differences in social demographic characteristics and VLs in the pre- and post-implementation periods. Factors from univariate analysis with *P* < 0.10 and/or those previously shown to be associated with the differences in social demographic characteristics and VLs were included in the multivariate regression models, adjusted odds ratios (AOR) were calculated along with coinciding 95% confidence intervals (CI). In order to mitigate the effects of missing VL data, SPSS MCMC was used to conduct multiple imputations for VLs on the log-transformed level based on the factors associated with the available VL data [7, 27]. We calculated mCVL and undetectable VL rates stratified by the demographic characteristics to assess differences in these characteristics in pre- and post-implementation periods. The pre- and post-implementation mCVLs were calculated and displayed at the county level in the ArcGIS 10.2. Significance was determined at *P*≤0.05 level and β = 0.1. Data were analyzed using SPSS version 19.0.

## Results

Since the implementation of the “Treatment as Prevention” strategy in Hangzhou, the ART coverage increased from 60.7% (839/1383) in January 2014 to 92.3% (2183/2365) in June 2016 (*P* < 0.001). A total of 940 HIV infected MSM were included in the study: 490 (52.1%) and 450 (47.9%) individuals in the pre- and post-implementation periods, respectively. The ART coverage was 51.0% (250/490) and 83.8% (377/450) in the pre- and post-implementation periods (*P* < 0.001). There were significant differences in marital status (*P* = 0.017), education levels (*P* < 0.001), and CD4 counts (*P* = 0.010) between individuals in the pre- and post-implementation periods. There were no significant differences in the other demographic characteristics investigated (*P* > 0.05). The multivariate logistic regression analysis showed that level of education (AOR =1.89, 95% CI 1.38–2.60) and CD4 counts (AOR = 2.16, 95% CI 1.30–3.61) were significantly higher in the pre- rather than post-implementation period (Table 1).

**Table 1:** Social demographic and characteristics of patients, pre- and post-implementation of a “Treatment as Prevention” strategy for HIV-infected men who have sex with men in Hangzhou

Variables	N (%)	Before implementation (2014) (%)	After implementation (2016) (%)	Univariate		Multivariate
				χ^2^	*P-*value	AOR(95% CI)
Marital status				8.12	0.017	
Single	628(66.1)	307(62.7)	321(71.3)			
Married/cohabitation	141(14.8)	81(16.5)	60(13.3)			
Divorced/separated	171(18.0)	102(20.8)	69(15.3)			
Ethnicity				0.01	0.946	
Han	923(97.2)	481(98.2)	442(98.2)			
Minority	17(1.8)	9(1.8)	8(1.8)			
Education				18.70	<0.001	
Junior high school and below	266(28.0)	161(32.9)	105(23.3)			1.00
High school and junior college	258(27.2)	144(29.4)	114(25.3)			1.20(0.85–1.71)
College	416(43.8)	185(37.8)	231(51.3)			1.89(1.38–2.60)
Age, years				2.98	0.395	
< 25	267(28.1)	131(26.7)	136(30.2)			
25–29	250(26.3)	126(25.7)	124(27.6)			
30–39	260(27.4)	141(28.8)	119(26.4)			
≥ 40	163(17.2)	92(18.8)	71(15.8)			
Follow-up				1.42	0.233	
Yes	841(88.5)	444(90.6)	397(88.2)			
No	99(10.4)	46(9.4)	53(11.8)			
CD4 count (cells/μL)				11.33	0.010	
0–199	81(8.5)	53(10.9)	28(5.9)			1.00
200–349	237(24.9)	129(26.5)	108(22.6)			1.58(0.93–2.68)
350–499	286(30.1)	153(31.4)	133(27.9)			1.67(0.99–2.81)
≥ 500	330(34.7)	152(31.2)	178(37.3)			2.16(1.30–3.61)
Registered residence						
Local	771(81.2)	407(83.1)	364(80.9)	0.75	0.386	
Nonlocal	169(17.8)	73(16.9)	86(19.1)			
ART				113.33	<0.001	–
Yes	627(66.7)	250(51.0)	377(83.8)			
No	313(33.3)	240(49.0)	73(16.2)			

^*^ Pre- and post-implementation, 3 patients were deficient in CD4 count

Acronyms: *χ*^2^ chi squared test, *AOR*, adjusted odds ratio, *CI* confidence interval

In total, 89.5% (841/940) of patients had data available on VL rates; there were no differences in available VL rates for those in the pre- (90.5%) and post-implementation (88.2%) periods (χ2=1.422,*P* = 0.233). HIV-infected MSM who did not receive ART (*P* < 0.001) and residents registered as nonlocals (*P* < 0.001) had more missing VL data than those who received ART and local residence. Multivariate logistic regression analysis demonstrated the following: those on ART had 4.81 (95% CI, 2.96–7.83) times the odds of having available VL data than those not on ART; nonlocal residents had 0.46 (95% CI, 0.28–0.74) times the odds of having available VL data than locals; those in the post-implementation period had 0.41 (95% CI, 0.25–0.67) times the odds of having available VL data than those treated in the post-implementation period (Table 2).

**Table 2:** Factors associated with available viral load data among HIV-infected men who have sex with men in Hangzhou

Variables	Total	VL data not available	VL data available	Univariate		Multivariate
				χ^2^	*P-*value	AOR(95% CI)
Overall	940	99(10.5)	841(89.5)			
Marital status				3.53	0.171	
Single	628	71(71.7)	557(66.2)			
Married/cohabitation	141	7(7.1)	134(15.9)			
Divorced/separated	171	21(21.2)	150(17.8)			
Ethnicity				2.04	0.153	
Han	923	99(100)	824(98.0)			
Minority	17	0(0)	17(2.0)			
Education				3.53	0.171	
Junior high school and below	266	24(24.2)	242(28.8)			
High school and junior college	258	35(35.4)	223(26.5)			
College	416	40(40.4)	376(44.7)			
Age, years				5.68	0.129	
< 25	267	37(37.4)	230(27.3)			
25–29	250	27(27.3)	223(26.5)			
30–39	260	23(23.2)	237(28.2)			
≥ 40	163	12(12.1)	151(18)			
CD4 count (cells/μL)				1.52	0.677	
0–199	81	8(8.6)	73(8.7)			
200–349	237	19(20.4)	218(25.9)			
350–499	286	32(34.4)	254(30.2)			
≥ 500	330	34(36.6)	296(35.2)			
Received ART				34.46	<0.001	
No	313	59(59.6)	254(30.2)			1.00
Yes	627	40(40.4)	587(69.8)			4.81(2.96–7.83)
Registered residence				13.24	<0.001	
Local	771	68(68.7)	703(83.6)			1.00
Nonlocal	169	31(31.3)	138(16.4)			0.46(0.28–0.74)
Study period				1.42	0.233	
Pre-intervention	490	46(46.5)	444(52.8)			1.00
Post-intervention	450	53(53.5)	397(47.2)			0.41(0.25–0.67)

* Pre- and post-implementation, 3 patients were deficient in CD4

Acronyms: *χ2*, chi squared test, *AOR* adjusted odds ratio, *CI* confidence interval, *ART* antiretroviral therapy

The mCVL in the pre-implementation period was 579 copies/mL; however, this decreased by 94.1% to 33 copies/mL in the post-implementation period (Kruskal-Wallis < 0.001). The median VL was 18,451 copies/mL (IQR 3110–83,502) and 10 copies/mL (IQR 10–10) in the pre- and post-implementation periods, respectively. The undetectable VL rate was 50.0% in the pre-implementation period; however, this increased to 84.7% in the post-implementation period (*P* < 0.001), (Table 3).

**Table 3:** Social demographic differences between the mean community viral load and undetectable viral loads rates pre- and post-implementation of a “Treatment as Prevention” strategy for HIV-infected men who have sex with men in Hangzhou

Variables	Pre-implementation					Post-implementation					Kruskal-Wallis^a^	VL≤400%^a^
	mCVL	VL median(IQR)	Kruskal-Wallis	VL≤400%	*P*-value	mCVL	VL median(IQR)	Kruskal-Wallis	VL≤400%	*P*-value		
Overall	579	18,451(3110–83,502)		50.0		33	10(10–10)		84.7		<0.001	<0.001
Marital status			<0.001		<0.001			0.024		0.007		
Single	1058	3000(10–41,002)		38.1		34	10(10–23)		81.3		<0.001	<0.001
Married/cohabitation	78	10(10–259)		77.8		17	10(10–10)		91.7		<0.001	0.027
Divorced/separated	150	25(10–4893)		63.7		12	10(10–10)		94.2		<0.001	<0.001
Ethnicity			0.071		0.093			0.201		0.225		
Han	434	350(10–19,002)		50.5		27	10(10–10)		84.4		<0.001	<0.001
Minority	6265	35,003(2105–75,499)		22.2		10	10(10–10)		100.0		0.003	0.001
Education			0.016		0.013			0.012		0.138		
Junior high school and below	234	55(10–9900)		59.0		17	10(10–10)		88.6		<0.001	<0.001
High school and junior college	475	620(10–29,677)		48.6		17	10(10–10)		87.7		<0.001	<0.001
College	795	1900(10–41,350)		43.2		38	10(10–108)		81.4		<0.001	<0.001
Age, years			<0.001		<0.001			0.135		0.031		
< 25	1049	2790(10–40,004)		34.4		41	10(10–195)		77.9		<0.001	<0.001
25–29	947	3100(10–33,027)		40.5		25	10(10–10)		85.5		<0.001	<0.001
30–39	360	160(10–22,849)		55.3		22	10(10–10)		86.6		<0.001	<0.001
≥ 40	84	10(10–10)		77.2		17	10(10–10)		93.0		<0.001	0.006
CD4 count (cells/μL)			0.943		0.215			0.034		0.349		
0–199	492	230(10–31,501)		54.7		99	10(10–1567)		75.0		0.043	0.074
200–349	515	140(10–28,051)		55.0		40	10(10–60)		83.3		<0.001	<0.001
350–499	509	350(10–15,999)		43.4		25	10(10–10)		86.5		<0.001	<0.001
≥ 500	739	1800(10–20,749)		50.1		28	10(10–10)		85.2		<0.001	<0.001
Received ART			<0.001		<0.001			<0.001		<0.001		
No	11,241	18,451(3110–83,502)		10.8		3476	4109(1165–19,902)		15.1		<0.001	0.326
Yes	32	10(10–43)		87.6		12	10(10–10)		98.1		<0.001	<0.001
Registered residence			0.055		0.041			0.022		0.164		
Local	408	206(10–19,999)		52.1		10	10(10–10)		83.5		<0.001	<0.001
Nonlocal	902	1900(25–24,998)		39.8		14	10(10–10)		84.7		<0.001	<0.001

^a^ Pre- versus post-implementation

Acronyms: *mCVL* mean community viral load, *VL* viral load, *χ2* chi squared test, *AOR* adjusted odds ratio, *CI* confidence interval, *ART*, antiretroviral therapy

There were significant differences in mCVL in the pre-implementation period based on marital status (*P* < 0.001), education (*P* = 0.016), age (*P* < 0.001) and treatment with ART (*P* < 0.001). There were also significant differences in the mCVL in the post-implementation period based on marital status (*P* = 0.007), education (*P* = 0.012), CD4 count (*P* = 0.034), treatment with ART (*P* < 0.001) and place of registered residence (*P* = 0.022). The mCVL decreased for all variables investigated post implementation of the treatment strategy (*P* < 0.05), (Table 3).

There were no significant differences in the undetectable VL rate in the pre-implementation period based on the variables investigated with the exception of ethnicity (*P* = 0.093) and CD4 count (*P* = 0.215); however, significant differences existed based on marital status (*P* = 0.007), age (*P* = 0.031), and treatment with ART (*P* < 0.001) in the post-implementation period. The undetectable VL rate increased for all variables investigated post implementation of the treatment strategy with the exception of CD4 count <200 cells/μL and those who never received ART. Those HIV-infected individuals who never received ART had an undetectable VL rate of 10.8% before implementation and this increased to 15.1% after implementation (*P* = 0.326), (Table 3).

**Fig. 1:**
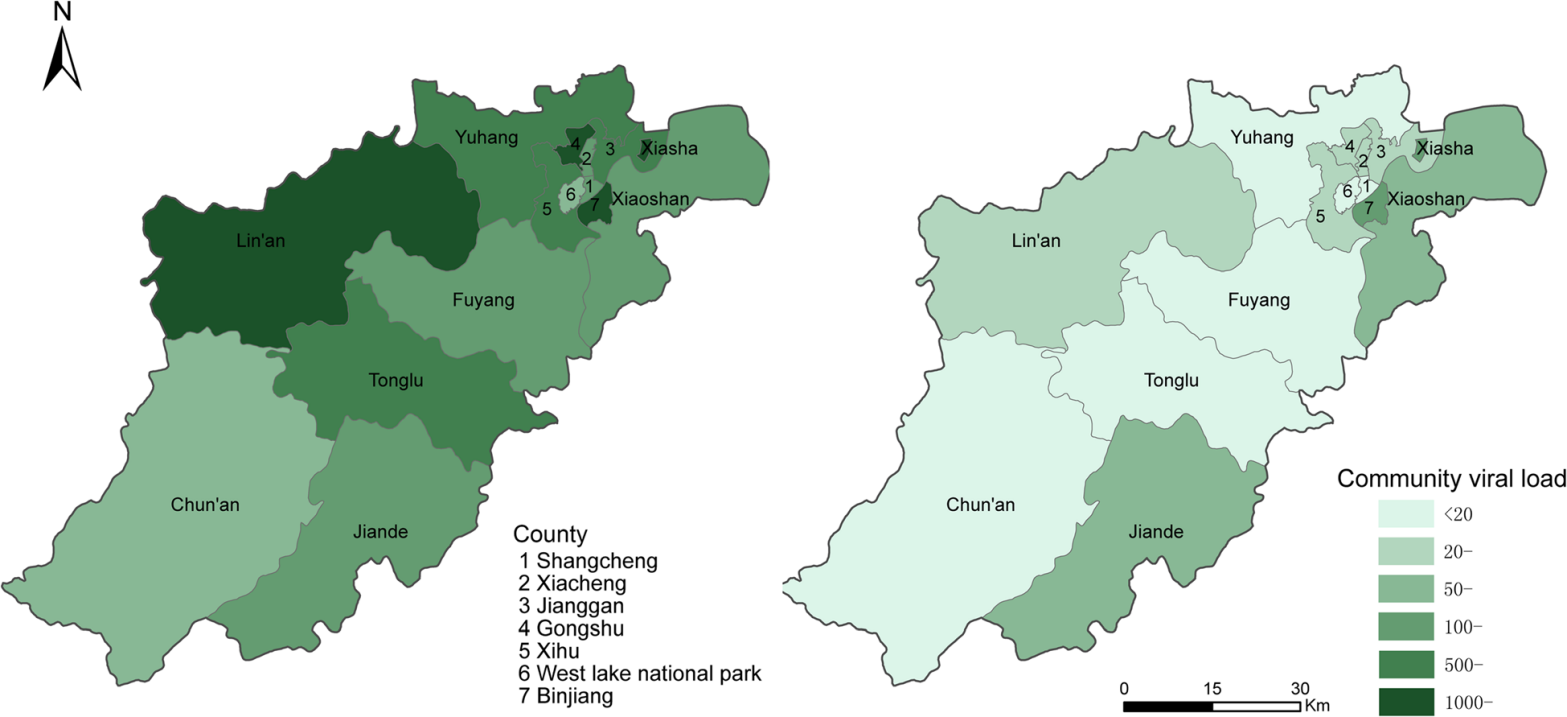
Mean community viral load of Hangzhou counties pre- and post-implementation of a “Treatment as Prevention” strategy for HIV-infected men who have sex with men

The mCVL at the county level of Hangzhou, analyzed according to the patient’s resident county, significantly decreased in each county (Kruskal-Wallis < 0.05) in the post-implementation period. (Fig. 1).

### HIV incidence and new HIV infection

The sentinel surveillance of MSM in Hangzhou showed the HIV incidence decreased from 5.71% (95% CI 2.36–9.07) to 3.58% (95% CI 0.38–6.78) in the pre- and post-implementation periods (*P* > 0.05). Using BED-CEIA methods it was determined that there were 77 newly infected individuals from January to June in 2014 and 73 newly infected individuals from January to June in 2016. Meanwhile, the frequency of patients with CD4 counts ≥600 cells/μL was 27 and 21 in the pre- and post-implementation periods, respectively.

## Discussion

Following the implementation of the “Treatment as Prevention” strategy in Hangzhou among HIV-infected MSM, the ART coverage increased from 60.7 to 92.3%, the mCVL decreased from 579 to 33 copies/mL, and the undetectable VL increased from 50.0 to 84.7%. Concurrently, the HIV incidence decreased from 5.71 to 3.58%. Despite this finding, the frequency of new HIV infections did not increase. There were differences in education levels and CD4 counts pre- and post- implementation of this strategy. The difference in CD4 counts in the pre- and post-implementation periods was mainly due to lack of ART that led to increased CD4 counts in the pre-implementation period; the intervention strategy was to encourage patients to receive ART. The rapid increase in the frequency of MSM in recent years in Hangzhou has occurred mainly among young, highly educated individuals. Therefore, the social demographic characteristics were balanced between the study periods.

The findings of this study are consistent with various other studies conducted in different countries. In a study conducted in New York [28], which included 7196 patients, the access to ART consistently increased from 78% in 2007, to 93% in 2014. Concurrently, virological suppression increased from 58 to 80%, and mean HIV RNA decreased from 351 to 73 copies/mL, from 2007 to 2014. Post implementation of a similar strategy in Rhode Island [13, 29], the ART coverage increased from 67 to 85%, VL suppression increased by 22%, and the mCVL decreased significantly; meanwhile, the number of newly diagnosed HIV infections decreased from 178 to 106, from 2004 to 2010. MSM are known to have multiple sexual partners and frequently have unprotected anal intercourse. Thus, ART may be less effective in preventing transmission among MSM, given the higher transmission probabilities associated with unprotected anal intercourse [5, 30]. Our study however, confirmed a population-level association between increased ART coverage and decreased mCVL; although there was not a significant decrease in the incidence and frequency of new HIV infections, the HIV incidence and frequency of new HIV infections did not continue to increase among MSM in Hangzhou.

Similar to the studies in San Francisco [7], Canada [18], South Carolina [16], and France [12], our study showed that increasing ART coverage decreased the CVL and reduced the frequency of new HIV infections. One study conducted in South Africa revealed that high ART coverage led to a substantial decrease in the rate of new HIV infections [30, 31]. Another large study from South Africa [32], showed a 1% increase in ART coverage was associated with a 1.4% decline in the risk of acquisition of new HIV infections. Similar to findings from South Africa, data from India [10] including 22,503 participants also identified an inverse association between population ART coverage and HIV incidence. Although the number of new HIV infections also declined, it was different from the above studies; the previous studies used the newly diagnosed infections to approximate new infections. We used different methods in our study to determine the number of new infections. A study conducted in Canada [20] arbitrarily used the CD4 cut-off of 350 cells/mm^3^ to indicate new versus old HIV infection. Moreover, several previous studies used new HIV diagnoses as a direct proxy for new infections [7, 12, 18, 31], even though the average lag time between HIV infection and diagnosis was substantial. Such studies fail to take into account that newly diagnosed infections can be affected by inadequate case-finding strategies [7, 18]. All newly diagnosed patients acquired HIV at some unknown earlier time, and so they were not “incident” in the traditional use of the word [33]. Recently it was estimated that nearly 40% of HIV-infected patients have delayed diagnosis (defined as a baseline CD4+ T cell <200 cells/μL) in China [22]; thus, using the new HIV diagnoses as new infections was unsuitable in our study. We therefore used BED-CEIA detection to determine new infections in order to reduce bias. In doing so, we were able to more reliably evaluate the mCVL and new HIV infections, thus accurately reflecting the impact of ART on new HIV infections.

The information on available VLs was distinctively different in the previous research studies; the proportion of patients with available VL data in San Francisco, New York, and Washington was 75% [7], 64% [9], and 48% [11], respectively. In the current study, when VLs were missing for more than 30% of patients, it would result in bias; therefore, multiple imputed VL data were used. VL data was available for 89.5% of individuals included in our study; therefore, we could better reflect the overall CVL at a population-level. Patients on ART had 4.81 times the odds of having available VL data when compared with those not on ART. These findings suggest that those who never start ART might have a higher frequency of missing data as they do not attend follow-up visits. Additionally, HIV-infected patients who never received ART had higher VLs; overall CVL might be underestimated due to the missing VL data [27]. A study conducted in Canada [34], including 719 HIV-infected MSM, showed that patients with a VL more than 200 copies/mL were more likely to have unprotected anal intercourse with a known HIV-negative or an unknown serostatus partner (AOR =3.13). These findings suggest that people with higher CD4 counts have a greater overall perceived wellness; targeted interventions should be implemented for untreated patients to reduce the community risk for transmission. Patients with CD4 counts lower than 200 cells/μL had no differences in the undetectable VL rates pre- and post-implementation of the strategy, potentially indicating that the transmission of HIV infection would not be reduced among patients with lower CD4 counts. Additional interventions are therefore needed for patients with lower CD4 counts who are at risk of poor clinical outcomes.

Our study was consistent with the findings from previous ecological studies that focused on associations between ART coverage and aggregate outcomes associated with CVL trends and the frequency of new HIV infections in a given geographical area [7, 18]. We found a significantly decreased mCVL at the county level following implementation of this treatment strategy. With the decline in mCVL, the HIV incidence and frequency of new HIV infections stopped increasing. Therefore, mCVL could be used as an indicator of HIV incidence [8, 10]. In India [10], which has a high prevalence of patients with high VLs, there is a high probability of coming into contact with an individual who could transmit HIV infection, compared with countries where large proportions of patients have low VLs. A meta-analysis [35] showed that in countries in sub-Saharan Africa (SSA), CVL appeared to be higher than in other regions; this phenomenon might be the central driver of the massive HIV epidemic in this region. Several studies have demonstrated that HIV incidence is significantly associated with HIV prevalence. Data from South Africa [32] revealed that an HIV uninfected individual was 2.2 times as likely to acquire HIV in a community where HIV prevalence was >25% relative to the base category of <10%. Therefore, when evaluating the association between increasing ART coverage and CVL, the CVL level, the undetectable VL rate, the local HIV epidemic, and the HIV prevalence should be taken into account. All these factors may affect the HIV incidence and the number of new infections in different countries or at different population-levels.

Another study conducted in South Africa [36] showed that among patients who did not receive ART, 17.2% had undetectable VLs; the data from San Francisco from 2004 to 2014 demonstrated that among MSM who were reported to have never received ART, undetectable VLs were found in 25% (5/19) in 2004, 18% (2/11) in 2008, and 43% (3/7) in 2011 [37]. A study from Uganda showed that 10% of untreated patients had undetectable VLs [38]. Consistent with the previous studies, a proportion of individuals without ART had undetectable VLs in our study. Blood samples from MSM who had never received ART were carefully checked; 10.8% and 15.2% pre- and post- implementation of the treatment strategy had undetectable VLs, respectively. Data from Uganda [39] showed that 1.4% of newly diagnosed HIV cases were controllers with continual undetectable VLs. Our study suggests that in the case of large-scale implementation of an ART strategy, patients should be informed during consent that nearly 10% of the infections have no detectable VLs before ART is initiated. Further guidance is needed on how to manage untreated patients with undetectable VLs. VL detection should be performed before ART is administered in order to better assess the efficacy of ART.

There were several limitations in our study. The strategy was only implemented in Hangzhou, according to the migratory patterns of MSM, because the migratory infections were introduced into Hangzhou over a short period of time. This led to the new infections occurring in the region while local infections were transmitted outside of the region. It would be not be valid to assess the HIV transmission based on the migratory patterns of MSM. A study in Canada [18] showed that migratory HIV populations might have produced a liberal bias. The transited HIV infection caused by migratory populations could not be estimated, particularly in Hangzhou, as it is a famous tourist city. Additionally, it could also not be estimated in the capital of Zhejiang province where the economy is more developed than other regions in China, because the MSM migrate frequently. Therefore, according to the migrant HIV-infected MSM, it was unable to evaluate the impact of increasing ART coverage on the HIV incidence and frequency of new HIV infections. Our study did not include the undiagnosed infections, as we were unable to assess the infections in undiagnosed individuals. Some research has revealed that persons unaware of their HIV infection might have higher VLs and/or high viral characteristics of acute/early infection [33, 37, 40]. The random sampling method used to select HIV-infected MSM were based on the patients’ ID numbers instead of if they ever received ART, thus the proportion of sampling treatment underestimated the proportion of actual treatment. Meanwhile, the ART coverage increased over the 2-year study period reaching 90%. The intervention strategies were implemented for a short period of time; thus, we could not assess the long-term impact on HIV incidence and the frequency of new HIV infections. Further research is needed to provide more information on these factors.

## Conclusion

In conclusion, after the implementation of this “Treatment as Prevention” strategy among HIV-infected MSM in Hangzhou, our study confirmed a population-level association between increased ART coverage and decreased mCVL. Overall, 84.7% of HIV-infected MSM had undetectable VLs and were therefore no longer infectious. Thus this strategy was effective in curbing the incidence and frequency of new HIV infections among MSM. The “Treatment as Prevention” strategy can be used to decrease HIV incidence and frequency of new infections, particularly among MSM.

## Abbreviations

χ2: Chi squared test
AOR: Adjusted odds ratio
ART: Antiretroviral therapy
CI: Confidence interval
CVL: Community viral load
mCVL: Mean community viral load
MSM: Who have sex with men
VL: Viral load
